# Effects of Yulin Tong Bu formula on modulating gut microbiota and fecal metabolite interactions in mice with polycystic ovary syndrome

**DOI:** 10.3389/fendo.2023.1122709

**Published:** 2023-02-06

**Authors:** Ya-Nan Su, Mei-Jiao Wang, Jun-Pu Yang, Xiang-Lu Wu, Min Xia, Mei-Hua Bao, Yu-Bin Ding, Qian Feng, Li-Juan Fu

**Affiliations:** ^1^ Department of Herbal Medicine, Chongqing Key Laboratory of Traditional Chinese Medicine for Prevention and Cure of Metabolic Diseases, School of traditional Chinese Medicine, Chongqing Medical University, Chongqing, China; ^2^ Joint International Research Laboratory of Reproduction and Development of the Ministry of Education of China, School of Public Health, Chongqing Medical University, Chongqing, China; ^3^ Department of Physiology, School of Basic Medicine, Chongqing Medical University, Chongqing, China; ^4^ Department of Gynecology, Chongqing Hospital of Traditional Chinese Medicine, Chongqing, China; ^5^ Department of Pharmacology, Academician Workstation, Changsha Medical University, Changsha, China; ^6^ Department of Obstetrics and Gynecology, Chongqing General Hospital, University of Chinese Academy of Sciences, Chongqing, China

**Keywords:** polycystic ovary syndrome, YLTB formula, gut microbiota, metabolites, ferulic acid

## Abstract

**Background:**

Polycystic ovarian syndrome (PCOS) is a common endocrine disorder characterized by hyperandrogenism, ovarian dysfunction and polycystic ovarian morphology. Gut microbiota dysbiosis and metabolite are associated with PCOS clinical parameters. Yulin Tong Bu formula (YLTB), a traditional Chinese medicine formula, has been recently indicated to be capable of ameliorating polycystic ovary symptoms and correcting abnormal glucose metabolism. However, the therapeutic mechanism of YLTB on PCOS has not been fully elucidated.

**Methods:**

A pseudo sterile mouse model was established during this four-day acclimatization phase by giving the animals an antibiotic cocktail to remove the gut microbiota. Here, the therapeutic effects of YLTB on PCOS were investigated using dehydroepiandrosterone plus high-fat diet-induced PCOS mice model. Female prepuberal mice were randomly divided into three groups; namely, the control group, PCOS group and YLTB (38.68 g·kg^-1^·day^-1^) group. To test whether this effect is associated with the gut microbiota, we performed 16S rRNA sequencing studies to analyze the fecal microbiota of mice. The relationships among metabolites, gut microbiota, and PCOS phenotypes were further explored by using Spearman correlation analysis. Then, the effect of metabolite ferulic acid was then validated in PCOS mice.

**Results:**

Our results showed that YLTB treatment ameliorated PCOS features (ovarian dysfunction, delayed glucose clearance, decreased insulin sensitivity, deregulation of glucolipid metabolism and hormones, etc.) and significantly attenuated PCOS gut microbiota dysbiosis. Spearman correlation analysis showed that metabolites such as ferulic acid and folic acid are negatively correlated with PCOS clinical parameters. The effect of ferulic acid was similar to that of YLTB. In addition, the bacterial species such as *Bacteroides dorei* and *Bacteroides fragilis* were found to be positively related to PCOS clinical parameters, using the association study analysis.

**Conclusion:**

These results suggest that YLTB treatment systematically regulates the interaction between the gut microbiota and the associated metabolites to ameliorate PCOS, providing a solid theoretical basis for further validation of YLTB effect on human PCOS trials.

## Graphical abstract

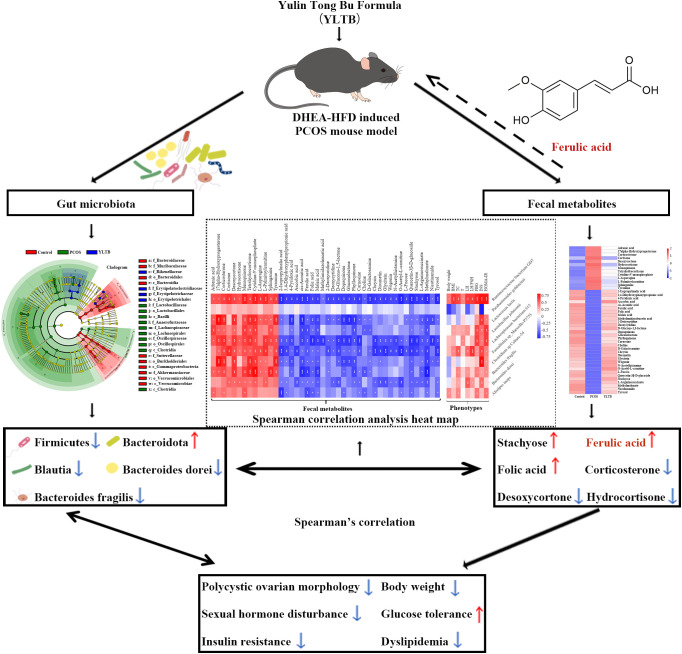

## 1 Introduction

Polycystic ovarian syndrome (PCOS) is a common endocrine disorder, from which 6-10% of reproductive females suffer severely (1, 2). The features of PCOS include hyperandrogenism, ovarian dysfunction and polycystic ovarian morphology (1–3). Usually, the disease is accompanied with insulin resistance (IR), obesity and metabolic disorders (4). Among metabolic disorders, dyslipidemia is characterized by increased levels of total cholesterol (TC), triglycerides (TG) and low-density lipoprotein cholesterol (LDL-C), but decreased levels of high-density lipoprotein cholesterol (HDL-C) (5).

The changes in those metabolites are often induced in human PCOS with high testosterone (T) levels (1) and obese (4) as well as gut microbiota disorder (6, 7). The gut microbiota is a complex ecosystem involved in host metabolic homeostasis, energy balance and immune modulation (8), which includes bacteria, fungi, viruses and protozoa (9). The diversity of gut microbiota can be divided into α or β diversity. The former refers to the species diversity within a community, and β diversity depicts the clustering of gut microbiota communities, mainly focusing the differences among different communities (10). The 16S rRNA sequencing studies have shown that disruption of the diversity and relative abundance of gut microbiota occurs during PCOS development (11). Gut microbiota dysbiosis is correlated with clinical parameters of PCOS women such as T, luteinizing hormone/follicle stimulating hormone (LH/FSH), LH, IR, fasting blood glucose (FBG), body mass index (BMI) and TG (12, 13). Fecal microbiota transplantation (FMT) of PCOS women into the germ-free mice led to variations in the levels of serum glucose, insulin (INS) and sex hormones (14). Consistently, increased Bacteroides vulgatus abundance resulted in ovarian dysfunction and metabolic disorders in the same germ-free mice (14).

In addition to those metabolites mentioned above, more have been reported to be associated with the gut microbiota of PCOS patients or rodent animals (14–16). For example, stachyose has a significantly negative correlation with *Ruminococcus 2* and regulates estrous cycle disorder, polycystic ovary morphology and T levels in PCOS rats (15). The levels of short-chain fatty acids (SCFAs) are decreased in PCOS rats (17), which play key functional regulatory roles in inflammation dissipation (18), IR and glucose tolerance (19). In addition, the levels of glyodeoxycholic acid (GDCA) and taurine deoxycholic acid (TUDCA) are negatively correlated with the abundance of *Bacteroides vulgatus* in the intestinal microbiota of PCOS patients (14).

The relationships among metabolites, gut microbiota, and PCOS sheds light on the development of novel treatments against PCOS. Currently, gonadotropins, clomiphene citrate and metformin have been clinically applied (20), but with many side effects such as ovarian hyperstimulation syndrome (21), drug resistance (22) and gastrointestinal distress (23, 24). Some of these could be avoided by using traditional Chinese medicine (TCM), such as FuFang ZhenZhu TiaoZhi formula (25), Bu Shen Hua Zhuo formula (26) and Liuwei Dihuang Pills (27). In TCM theory, the key pathogenesis of PCOS is based on “phlegm-dampness block and spleen-kidney deficiency”. Based on Buzhong Yiqi Decoction, which derived from Fu Qing Zhu Nv Ke and the key pathogenesis of PCOS, we created a decoction named as Yulin Tong Bu formula (YLTB). Our research team has been using the YLTB formula in the clinical treatment of PCOS for many years, and earlier clinical trials have shown that YLTB formula could improve the therapeutic effect of metformin (28). YLTB is made of 12 traditional Chinese herbs (listed in **Table 1**). Out of them, Astragalus mongholicus Bunge, Actaea cimicifuga L., Citrus × aurantium L. and Poria cocos (Schw.)Wolf were reported to impact metabolite levels and gut microbiota (29–33). For example, Citrus × aurantium L., mainly containing nobiletin, could improve high-fat diet (HFD)-induced obesity, deregulate intestinal lipid metabolism, and reshape gut microbiota (29, 30). Astragalus mongholicus Bunge includes soyasaponin I and formononetin, which can reshape gut microbiota (34), inhibit obesity and dyslipidemia, reduce IR and improve glucose homeostasis (35–37). Although these studies suggest the potential therapeutic effect of YLTB against PCOS, the molecular mechanism underlying it remains unclear.

**Table 1:** The Chinese herb drugs contained in YLTB.

Chinese name	Botanical name	Family	Genus	Part used	Weight (g)
Dangshen	Codonopsis pilosula (Franch.) Nannf.	Campanulaceae	Codonopsis Wall.	root	45
Huangqi	Astragalus mongholicus Bunge	Fabaceae	Astragalus L.	root	30
Baizhu	Atractylodes macrocephala Koidz.	Asteraceae	Atractylodes DC.	rhizome	30
Shengma	Actaea cimicifuga L.	Ranunculaceae	Cimicifuga L.	rhizome	10
Chenpi	Citrus × aurantium L.	Rutaceae	Citrus	fruit peel	10
Fuling	Poria cocos(Schw.)Wolf	Polyporaceae	Poria	sclerotium	15
Banxia	Pinellia ternata (Thunb.) Makino	Araceae	Pinellia Tenore	tuber	10
Cangzhu	Atractylodes lancea (Thunb.) DC.	Asteraceae	Atractylodes	rhizome	30
Jixueteng	Spatholobus suberectus Dunn	Fabaceae	Spatholobus Hassk.	stem	30
Xiangfu	Cyperus rotundus L.	Cyperaceae	Cyperus L.	rhizome	10
Tusizi	Cuscuta chinensis Lam.	Convolvulaceae	Subg. Grammica	ripe seed	20
Bajitian	Gynochthodes officinalis (F.C.How) Razafim. & B.Bremer	Rubiaceae	Morinda	root	15

In this study, we determined the therapeutic effect of YLTB on PCOS using dehydroepiandrosterone (DHEA) plus HFD-induced PCOS mice model. The mechanism could be explained by the ability of YLTB to orchestrate gut microbiota and the related metabolites. Compared with the control mice, PCOS mice presented a series of syndromes such as ovarian dysfunction, delayed glucose clearance, decreased insulin sensitivity, and metabolic deregulation of lipid as well as hormones. Those syndromes in PCOS mice can be greatly ameliorated by YLTB treatment. The 16S rRNA sequencing analysis was applied to demonstrate the landscape of gut microbial composition in PCOS mice. We found that the dysbiosis of PCOS gut microbiota was significantly attenuated by YLTB treatment. The correlation between gut microbiota and fecal metabolites in mice was further investigated by the association study analysis, combined with LC−MS (liquid chromatography-mass sectromety)-based metabolic sequencing analysis. The analysis results predicted that metabolites such as ferulic acid, folic acid, menaquinone and phylloquinone are negatively correlated with bacterial species such as *Bacteroides dorei* and *Bacteroides fragilis*. These bacteria were positively correlated with the PCOS clinical parameters such as TC, LH, FBG, and insulin resistance index in the homeostasis model (HOMA-IR). In addition, the therapeutic effect of ferulic acid was validated in PCOS mice, similar to YLTB effect. Taken together, YLTB treatment systematically regulate the interaction between gut microbiota and the associated metabolites to ameliorate PCOS.

## 2 Materials and methods

### 2.1 Preparation of Yulin Tong Bu formula

The herb details of YLTB are presented in **Table 1**. The clinical dose of YLTB was 255g (28), 45 g Dangshen (*Codonopsis pilosula (Franch.) Nannf.*), 30 g Huangqi (*Astragalus mongholicus Bunge*), 30 g Baizhu (*Atractylodes macrocephala Koidz.*), 10 g Shengma (*Actaea cimicifuga L.*), 10 g Chenpi (*Citrus × aurantium L.*), 15 g Fuling *(Poria Cocos (Schw.) Wolf.*), 10 g Banxia (*Pinellia ternata (Thunb.) Makino*), 30 g Cangzhu (*Atractylodes Lancea (Thunb.) Dc*.), 30 g Jixueteng (*Spatholobus Suberectus Dunn*), 10 g Xiangfu (*Cyperus rotundus L.*), 20 g Tusizi (*Cuscuta chinensis Lam.*), and 15 g Bajitian (*Gynochthodes officinalis (F.C.How) Razafim. & B.Bremer*), respectively. The Chinese herbs were purchased from Chongqing Hospital of Traditional Chinese Medicine. They were then suspended in distilled water at room temperature for one hour to soften the raw materials and facilitate the extraction of water-soluble ingredients in subsequent steps. The herbs were boiled for two hours and passed through filter paper. The process lasted two times followed by concentrating a decoction at 100°C, to obtain the extracts of YLTB containing 2.55-g raw herbs/mL. In this paper, the dosages of YLTB used in mice were converted from clinical dosage using the following equation (38, 39):


Dm=Dh/W × F

Where Dm is the administrated dose of YLTB for mouse in the present work, Dh is the clinical dose of YLTB, W is the weight of human body, F is the dose conversion factor of mouse and human. W is set as 60 kg. Considering the dose conversion factor of 9.1 between mouse and human, the high dosage is 38.68 g/kg, the medium dosage is 19.34 g/kg, and the low dosage is 9.67 g/kg.

### 2.2 Liquid chromatography–mass spectrometry

ACQUITY UPLC HSS T3 (2.1 × 100 mm 1.8 m columns) was used for liquid chromatography–mass spectrometry (LC-MS) (Waters, UPLC; Thermo, Q Exactive). The following chromatographic separation conditions were used: column temperature, 40°C; flow rate, 0.30 mL/min; mobile phase A, water + 0.05 percent formic acid; mobile phase B, acetonitrile; injection volume, 2 μL; automatic injector temperature, 4°C. The MS parameters are as follow: ESI+: spray voltage, 3.0 (ESI+) or 3.2 (ESI-) kV; S-Lens RF level, 30% (ESI+) or 60% (ESI-); heater temperature, 300 °C; sheath gas flow rate, 45 arb; auxiliary gas flow rate, 15 arb; sweep gas flow rate, 1arb; capillary temperature, 350°C. Scan duration is 100 ms, interscan time is 50 ms, and the scan range is 70-1050 m/z. Data was analyzed using Compound Discoverer 3.1 software (Thermo Fisher Scientific,USA), normalized, and converted into a two-dimensional matrix using Excel 2010 software, containing retention time, compound molecular weight, observations (samples), and peak intensity (39, 40).

### 2.3 Animals and treatment

Female prepuberal C57BL/6 (21-d-old) mice (specific pathogen-free, Animal Qualification Certificate No.2022109) were purchased from the Animal Management Center of Chongqing Medical University. The mice were maintained under standard circumstances (22-24 °C, 50% relative humidity, 12-hour dark/light cycle) with unrestricted access to food and water. The Institutional Animal Care and Use Committee of Chongqing Medical University authorized all mouse experimental procedures (Chongqing, China). Mice were given four days to adjust to their new surroundings before being tested. A pseudo sterile mouse model was established during this four-day acclimatization phase by giving the animals an antibiotic cocktail (ABX) to remove the gut microbiota. Freshly prepared antibiotic cocktail comprised 1 mg mL^−1^ ampicillin sodium (1146GR005, Biofroxx, Einhausen, Germany), 1 mg mL^−1^ neomycin sulfate (N814740-5G, MACKUN, Shanghai, China), 1 mg mL^−1^ metronidazole (M1547-5G, Sigma−Aldrich, MO, USA), and 0.5 mg mL^−1^ vancomycin hydrochloride (#1161GR1001, Biofroxx, Einhausen, Germany) in drinking water, and the mice were given autoclaved drinking water four days later.

According to the preliminary experiment (**Figure S1**), 30 mice were randomly divided into six groups (n = 5) to optimize the dosage of YLTB: control group, PCOS group, high dosage YLTB group [YLTB (H), 38.68 g·kg^-1^·day^-1^], medium dosage YLTB group [YLTB (M), 19.34 g·kg^-1^·day^-1^], low dosage YLTB group [YLTB (L), 9.67 g kg^-1^·day^-1^], and metformin group (positive control, 250 mg kg^-1^·day^-1^, #D150959-5G, Sigma−Aldrich, MO, USA). To establish the PCOS group, mice were fed a high-fat diet (HFD, 60% kcal% fat; #D12492, Research Diets, Inc., NB, USA) and received daily subcutaneous injections of DHEA (#252805-10GM, Millipore, CA, USA; 60 mg/kg, dissolved in 0.1 ml of sesame oil) (41, 42). To determine the most effective YLTB dosage for PCOS, mice were fed an HFD, gavaged with various YLTB dosages, and injected with DHEA on the same day. The control group was fed a normal chow diet (NCD) and injected daily with sesame oil (#8008-74-0, Acros, BE, USA).

In order to verify the effects of high dose YLTB group on glucolipid metabolism, intestinal microbiota and fecal metabolism of PCOS mice, 30 mice were randomly divided into three groups (n = 10), namely, the control group, PCOS group and YLTB group (38.68 g·kg^-1^·day^-1^). To test the efficacy of the metabolite ferulic acid, 40 mice were randomly divided into four groups (n = 10), namely, the control group, PCOS group, FA1 group (50 mg·kg^-1^·day^-1^) and FA2 group (100 mg·kg^-1^·day^-1^) (33, 43). For FA groups, mice were fed an HFD and subcutaneously injected with DHEA daily, as well as administered ferulic acid (purity > 99.00%, #128708, Sigma−Aldrich, MO, USA, dissolved in lukewarm water) by oral gavage every day.

The mice mentioned above were all treated for 20 days. At the end of the experiment, mice were fasted over 12 hours and anesthetized by intraperitoneal injection of 10% chloral hydrate with 0.1 mL/10 g, and then weighed, measured to determine the distance from the tip of the nose to the anus, and tested for fasting blood glucose and oral glucose tolerance tests (OGTTs). The serum was collected and centrifuged for 20 minutes at 3,000 rpm to measure insulin and sex hormones. The ovaries and uteri of mice were collected, weighed, and wax-fixed or snap-frozen and stored at –80 °C for further use.

### 2.4 Estrous cycle determination

From the 11th to the 20th day of the gavage treatment, vaginal smears of mice were collected every day. The estrous cycle stage was determined by microscopic analysis of the predominant cell type in vaginal smears.

### 2.5 Measurement of serum biochemical markers

Enzyme-linked immunosorbent assay (ELISA) kits were used to determine serum testosterone, luteinizing hormone (LH), follicle stimulating hormone (FSH) and serum insulin levels (#JL25196, #JL10432, #JL10329, #JL11459, Shanghai Jianglai Biology, Shanghai, China). A glucometer was used to evaluate blood glucose. The homeostatic model assessment of IR (HOMA-IR) index was calculated using the formula: [FBG (mmol/L)] × [FINS (lU/mL)]/22.5. After an overnight fast, the oral glucose tolerance tests (OGTTs) were administered *via* oral gavage of 2 g/kg glucose. The blood glucose level of the mice was tested using blood glucose test paper at 0, 15, 30, 60, 90, and 120 minutes. At least 5 mice were used for these experiments.

### 2.6 Body weight, Lee’s index analysis, BMI and lipid profile analysis

Every four days, the mice weight was recorded. The length from the tip of the nose to the anus of the mouse was measured after anesthesia, and the Lee’s index and BMI were calculated (Lee’s index = [Body mass (g) × 1000]^1/3^/body length (cm), BMI=body weight (kg)/body length (m^2^)). Biochemical analysis kits were used to assess serum lipid profiles, including TC, TG, HDL-C, and LDL-C (#A111-1-1, #A110-1-1, #A112-1-1, #A113-1-1; Nanjing Jiancheng Bioengineering Institute, Nanjing, China).

### 2.7 Tissue processing and H&E staining

Mouse ovaries were harvested, fixed with 4% paraformaldehyde (#BL539A, Biosharp) and embedded in paraffin. Hematoxylin and eosin (H&E) kits were used to stain ovary sections to observe follicle changes. Sections of 5 μm were placed on glass slides, with 40 μm discarded between each section; six sections from each ovary were collected. These tests required at least 5 mice per group (44, 45).

### 2.8 16S rRNA sequencing

The fecal pellets were collected in sterile cryopreservation tubes immediately after being discharged before sacrifice. Before analysis, the fecal samples were snap-frozen and stored at –80 °C. Fecal samples were sent to Novogene (Beijing, China) for 16S RNA sequencing. DNA was extracted from mouse fecal samples using a Magnetic Soil and Stool DNA Kit (#DP712, Tiangen). DNA concentration and purity were measured on 1% agarose gels. DNA was diluted to 1 ng/μL in sterile water according to the concentration.

The V3–V4 hypervariable region of the 16S rRNA gene was amplified by PCR at 98 °C for 1 minute, followed by 30 cycles of denaturation at 98 °C for 10 seconds, annealing at 50 °C for 30 seconds, elongation at 72 °C for 30 seconds, and final incubation at 72 °C for 5 minutes. The common primer pair (515F5′-CCTAYGGGRBGCASCAG-3’; 806R5′-GGACTACNNGGGTATCTAAT-3′) was used to amplify the bacterial 16S rRNA gene. All PCRs were carried out with 15 µL of Phusion^®^ High-Fidelity PCR Master Mix (#M0531S, New England Biolabs, MA, USA), 2 μM primers, and 10 ng template DNA. The amplification products were isolated using a 2% agarose gel and purified with a Universal DNA Purification Kit (#DP214, Tiangen, Beijing, China). The NEB Next^®^ Ultra DNA Library Prep Kit (#E7370L, Illumina, CA, USA) was used for library construction. The established library was detected by an Agilent Bioanalyzer 5400 system and quantified by Q-PCR. Finally, the library was sequenced on an Illumina NovaSeq platform (Illumina Novaseq6000, Illumina, CA, USA), and 250 bp paired-end reads were generated (46).

### 2.9 Fecal metabolic profiling

Individual mouse feces samples (100 mg) were crushed in liquid nitrogen before being resuspended in prechilled 80% methanol using a well vortex (n = 6). The samples were placed on ice for 5 minutes before being centrifuged at 15,000 g and 4 °C for 20 minutes. LC−MS grade water was used to dilute some of the supernatant to a final concentration of 53% methanol. The samples were then transferred to a new tube and centrifuged at 15,000 g and 4 °C for 20 minutes. The supernatant was then injected into the LC−MS/MS apparatus for analysis.

UHPLC−MS/MS analyses were carried out using a Vanquish UHPLC system (ThermoFisher) paired with an Orbitrap Q ExactiveTM HF mass spectrometer (Thermo Fisher) by Novogene. Using a 17-min linear gradient at a flow rate of 0.2 mL/min, samples were injected onto a Hypersil Gold column (100 × 2.1 mm, 1.9 μm). Eluent A (0.1% formic acid in water) and eluent B (methanol) served as the eluents for the positive polarity mode. Eluent A (5 mM ammonium acetate, pH 9.0) and eluent B (methanol) were the eluents for the negative polarity mode. The solvent gradient was set as follows: 2% B, 1.5 min; 2-85% B, 3 min; 100% B, 10 min; 100-2% B, 10.1 min; 2% B, 12 min. The Q ExactiveTM HF mass spectrometer was operated in positive/negative polarity mode, with a spray voltage of 3.5 kV, capillary temperature at 320°C, sheath gas flow rate at 35 arb and aux gas flow rate at 10 arb, S-lens RF level at 60 and aux gas heater temperature at 350°C.

Compound Discoverer 3.1 (CD3.1, ThermoFisher) was used to analyze the raw data files produced by UHPLC−MS/MS to perform peak alignment, peak selection, and quantification for each metabolite. The primary parameters were established: retention time tolerance, 0.2 minutes; actual mass tolerance, 5 ppm; signal intensity tolerance, 30%; signal/noise ratio, 3; and minimum intensity. Peak intensities were then normalized to reflect the total spectral intensity. Based on additive ions, molecular ion peaks, and fragment ions, the normalized data were utilized to predict the molecular formula. To obtain correct qualitative and relative quantitative results, peaks were matched with the MassList, mzCloud (https://www.mzcloud.org/), and mzVault databases (46).

Statistical analyses were carried out using the statistical tools R (R version R-3.4.3), Python (Python 2.7.6 version) and CentOS (CentOS release 6.6). The KEGG, HMDB, and LIPIDMaps databases were used to annotate these metabolites. MetaX was used to perform partial least squares discriminant analysis (PLS-DA) and principal components analysis (PCA). Univariate analysis (t test) was used to determine statistical significance (*P* value). VIP > 1 and *P* value < 0.05, as well as fold change ≥ 2 or FC ≤ 0.5, were deemed to indicate differential metabolites. The data for clustering heatmaps were standardized using z scores of differential metabolite intensity areas and plotted using the Pheatmap package in R language.

### 2.10 Data and statistical analysis

GraphPad Prism version 8.3 was used to analyze all data, which were then displayed as the means ± standard error of the mean (SEM). When *P* < 0.05, the results were considered statistically significant. Each variable was tested for differences among three or more groups using one-way or two-way analysis of variance (ANOVA) followed by Tukey’s multiple comparisons test to assess statistical significance. The cor.test function from the stats R package was used to perform a Spearman correlation among the levels of fecal metabolites, phenotype, and relative abundance of species. We only performed the correlation for those species (*P* < 0.05), phenotypes (*P* < 0.05) and metabolites (*P* < 0.05 VIP>1) that were statistically significant across groups.

## 3 Results

### 3.1 Chemical composition and LC-MS analysis of YLTB

**Figure 1:**
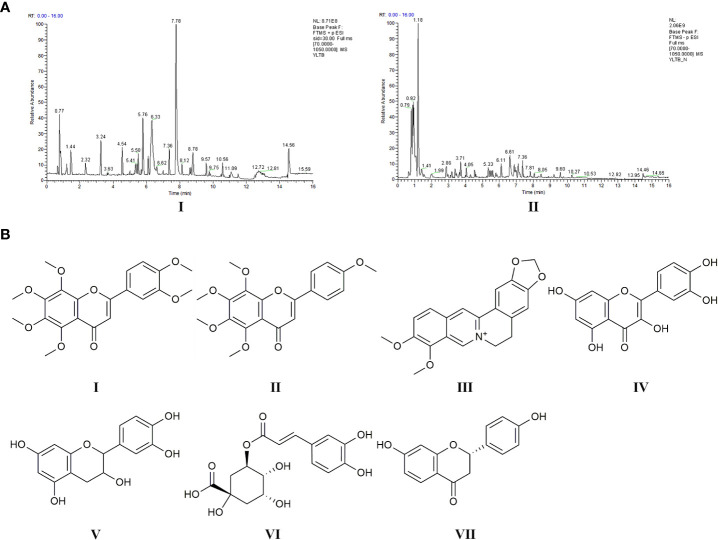
Chemical ingredients and LC-MS analysis of YLTB. **(A)** The total ion flow diagram for YLTB. I. Positive ion mode. II. Negative ion mode. **(B)** The molecular structures of seven important compounds identified in the analysis. I Nobiletin. II. Tangeritin. III. Berberine. IV. Quercetin. V;.Catechin. VI. Chlorogenic acid. VII. Liquiritigenin. RT, Run Time.

LC-MS analysis data of YLTB was performed using feature extraction and preprocessing with Compound Discoverer 3.1 software, normalized, and edited into a two-dimensional matrix using Excel 2010 software, including retention time, compound molecular weight, observations (samples), peak intensity and library matching (based on mzCloud and mzVault database, the mzVault include OTCML, KEGG and Chemspider database). The aim of this method was to complete the target screening without standard products. Several ingredients in YLTB were identified (**Figure 1A**, **Table 2** and **3**). The following seven important compounds were distinguishable, as shown in **Figure 1B**: (I) Nobiletin, (II) Tangeritin, (III) Berberine, (IV) Quercetin, (V) Catechin, (VI) Chlorogenic acid, and (VII) Liquiritigenin.

**Table 2:** LC-MS analysis data and the top 20 primary elemental composition of YLTB in positive ion mode.

NO.	Name	Formula	RT. [min]+	Molecular. Weight	Area
1	Benzothiazole	C7 H5 N S	6.302	135.01431	4452592429
2	Choline	C5 H13 N O	0.766	103.10016	1169277665
3	dimethyl(tetradecyl)amine	C16 H35 N	8.777	241.27692	1126416722
4	2-Mercaptobenzothiazole	C7 H5 N S2	6.603	166.98631	746968672
5	Creatinine	C4 H7 N3 O	0.9	113.05924	406383888.3
6	Maltol	C6 H6 O3	3.219	126.0319	380600348.2
7	1-Aminocyclopropane-1-carboxylic acid	C4 H7 N O2	0.821	101.04828	371648740.5
8	Pelargonidin	C15 H10 O5	5.422	270.05263	360361721.4
9	Nobiletin	C21 H22 O8	8.127	402.13114	352054134.2
10	DL-Stachydrine	C7 H13 N O2	0.84	143.09462	291876915.1
11	Tangeritin	C20 H20 O7	8.668	372.12019	290269427.2
12	Berberine	C20 H17 N O4	5.243	335.1147	275753582.5
13	Apocynin	C9 H10 O3	5.773	166.06301	229918577.4
14	7-Hydroxycoumarine	C9 H6 O3	3.634	162.0318	218666113.6
15	Indole	C8 H7 N	3.236	117.0582	206668891
16	3-Formylindole	C9 H7 N O	3.212	145.05278	206103311.7
17	PC (16:0/0:0)	C24 H50 N O7 P	10.111	495.3324	200782893.9
18	Pyrrole-2-carboxylic acid	C5 H5 N O2	1.2	111.03239	198819312.2
19	L-Pyroglutamic acid	C5 H7 N O3	1.195	129.04276	189601241.9
20	Quercetin	C15 H10 O7	4.597	302.04247	154436644.8

**Table 3:** LC-MS analysis data and the top 20 primary elemental composition of YLTB in negative ion mode.

NO.	MS2.name	Formula	RT.[min]−	Molecular.Weight	Area
1	D-FRUCTOSE	C6 H12 O6	0.796	180.06215	9655427837
2	α, α-Trehalose	C12 H22 O11	0.8	342.11478	7276775260
3	Citric acid	C6 H8 O7	1.182	192.02568	6221487818
4	2-Mercaptobenzothiazole	C7 H5 N S2	6.609	166.98485	2777846346
5	Gluconic acid	C6 H12 O7	0.788	196.05702	2316575258
6	D-(-)-Quinic acid	C7 H12 O6	0.83	192.06227	1234943274
7	Piscidic Acid	C11 H12 O7	2.848	256.05742	885987564.1
8	Catechin	C15 H14 O6	3.711	290.07826	846282140.4
9	L-Iditol	C6 H14 O6	0.779	182.07755	429101979.4
10	Chlorogenic acid	C16 H18 O9	3.18	354.09404	420580341.2
11	3-Hydroxy-3-methylglutaric acid	C6 H10 O5	1.547	162.05134	383522438.1
12	(-)-Gallocatechin	C15 H14 O7	2.946	306.07313	339562782.9
13	Quercetin-3β-D-glucoside	C21 H20 O12	4.601	464.0938	319376854.6
14	Formononetin-7-O-glucoside	C22 H22 O9	5.51	430.12437	311798194.2
15	(+/-)9-HpODE	C18 H32 O4	8.444	312.22923	276336157.3
16	Liquiritigenin	C15 H12 O4	7.16	256.07265	275731693.6
17	L-Arginine	C6 H14 N4 O2	0.717	174.1104	264866724.1
18	Jasmonic acid	C12 H18 O3	7.204	210.1244	253463816.8
19	Quinic acid	C7 H12 O6	2.318	192.06208	232639631.6
20	Procyanidin B2	C30 H26 O12	3.784	578.14076	220607671.2

### 3.2 YLTB formula attenuates ovarian dysfunction in DHEA plus HFD-induced PCOS mice

**Figure 2:**
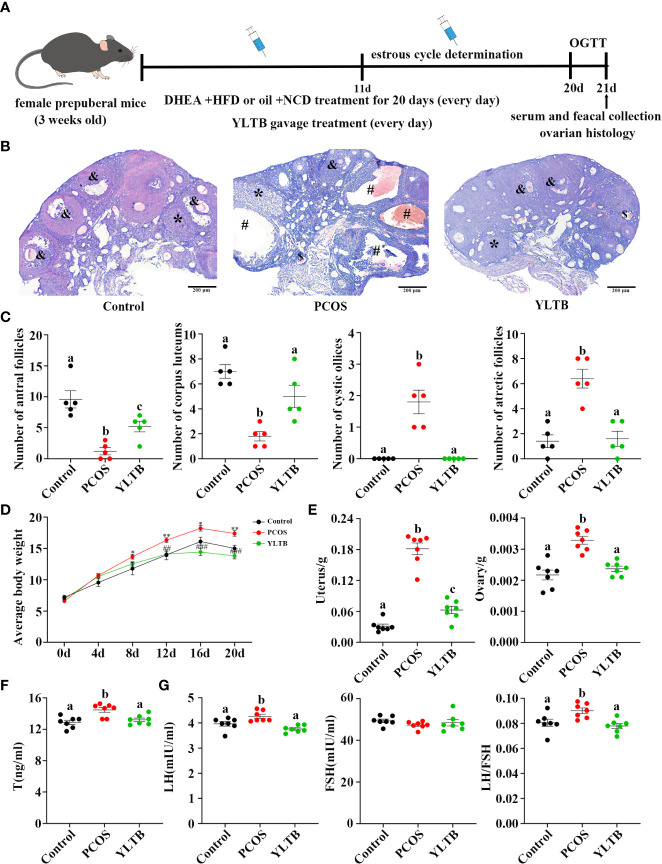
YLTB rescued ovarian dysfunction in PCOS mice. **(A)** The timeline of the experimental process. **(B)** Representative ovarian slices stained with hematoxylin and eosin (H&E). Scale bar = 200 μm; & indicates antral follicle; * indicates corpora luteum; # indicates cystic follicle; $ indicates atretic follicle. **(C)** The difference in the number of antral follicles, corpora lutea, cystic follicles, and atretic follicles among groups (n = 5/group). **(D)** Measurement of mouse body weight every four days among the three groups (n = 7/group, * P < 0.05; ** P < 0.01 compared with the control group, ## P < 0.01; ### P <0.001 compared with the PCOS group). **(E)** Measurement of the wet weight of the uterus and ovary among the three groups (n = 7/group). **(F, G)** Serum T, LH, and FSH levels were measured using an enzyme-linked immunosorbent assay kit, and the LH/FSH ratio was calculated (n = 7/group). Statistical significance was determined using one-way or two-way ANOVA with Tukey’s multiple comparisons test, and data are presented as the mean ± standard error of the mean (SEM). a, b and c indicate *P* < 0.05; if 2 groups have the same letter, it indicates no statistical significance.

Before the effect of YLTB on PCOS was investigated, the PCOS mice were generated by a HFD and DHEA subcutaneous injections (60 mg/kg body weight) for 20 days. The YLTB group mice were additionally gavaged with YLTB (38.68 g·kg^-1^·day^-1^) at the same time. The usage of YLTB was optimized in our preliminary data, as shown in the **Supplement Figure S1**. The control group was only daily injected with sesame oil. The mouse intervention process was illustrated in the **Figure 2A**. To address the effect of YLTB on mice ovary function, multiple parameters were evaluated, including ovarian morphology, mice weight, wet weight of uterus and ovaries, and sex hormone changes.

Ovarian H&E staining were performed to ensure a successful PCOS establishment and to determine YLTB’s effect on ovarian morphology as well as follicle counts (**Figure 2B**). Many cystic and atretic follicles in the ovaries of the PCOS group were absent in the ovaries of the YLTB group. The PCOS group also showed fewer antral follicles and corpus luteum, while YLTB administration increased them (**Figure 2C**). **Figure 2D** illustrated that the mice body weight was significantly decreased in the YLTB group, compared to the PCOS group. There was no significant difference in mice body weight between the control group and YLTB group. The uterus and ovary weights of PCOS mice were also significantly reduced after YLTB therapy (**Figure 2E**). In addition, YLTB dramatically downregulated serum T and LH levels in PCOS mice. Consistently, the LH/FSH ratio was significantly lower in the YLTB group in comparison to the PCOS group (**Figures 2F, G**). In summary, the application of YLTB largely restored these ovarian parameters of the PCOS group to the normal levels. Although it could affect sex hormone levels, YLTB did not impact the estrus cycle of the PCOS group (**Figure S2 A, B**).

### 3.3 YLTB ameliorates the disorder of glucose and lipid metabolism in PCOS mice

**Figure 3:**
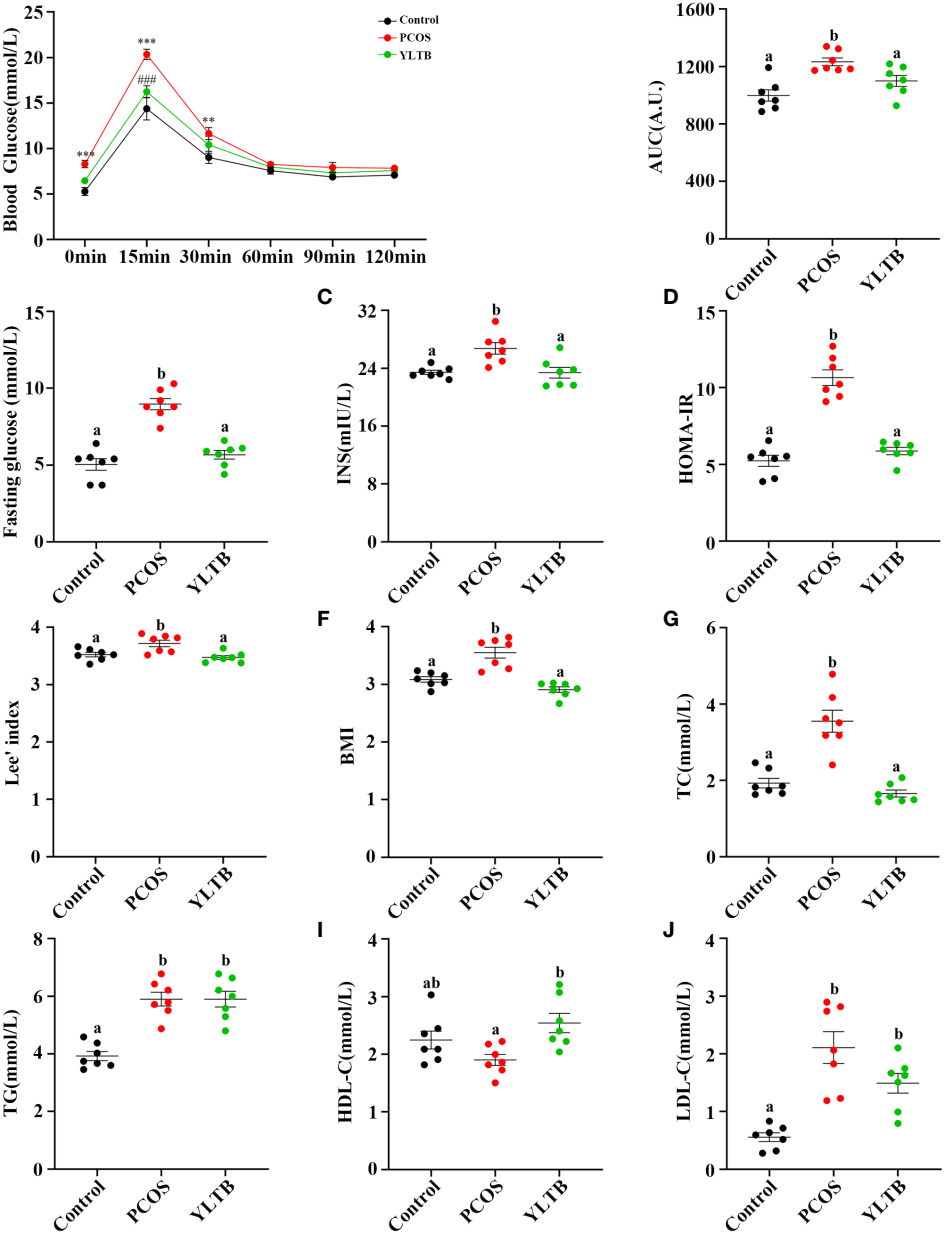
The effects of YLTB on glucose tolerance, insulin sensitivity and lipid metabolism in PCOS mice. **(A)** OGTTs in mice from the control, PCOS, and YLTB groups. The corresponding area under the curve (AUC) values of blood glucose levels in each group were calculated (** P < 0.01; *** P < 0.001 compared with the control group, ### P < 0.001 compared with the PCOS group). **(B, C)** Blood glucose and serum insulin level assessment after 12 h of fasting in mice from the control, PCOS, and YLTB (38.68 g·kg^-1^·day^-1^) groups. **(D)** The homeostasis model assessment of insulin resistance (HOMA-IR) index = [FBG (mmol/L)] × [FINS (lU/mL)]/22.5 in mice from the control, PCOS, and YLTB groups. **(E, F)** Lee’s index = [Body mass (g) × 1,000]^1/3^/body length (cm) and Body mass index (BMI = weight (kg)/height (m^2^) calculation. **(G–J)** Detection of TC, TG, HDL-C and LDL-C to evaluate the level of serum lipid metabolism in mice from the control, PCOS, and YLTB groups. n = 7/group, statistical significance was determined using one-way or two-way ANOVA with Tukey’s multiple comparisons test, and data are presented as the mean ± SEM. a and b indicate *P* < 0.05; if 2 groups have the same letter, it indicates no statistical significance.

To address whether YLTB ameliorates PCOS condition through alternation of glucose and lipid metabolism, the glucose levels, insulin levels, Lee’s index, BMI, lipid levels, and insulin sensitivity as well as the degree of obesity among the mice groups of the control, PCOS and YLTB were compared. Firstly, OGTTs were performed in those mice administered *via* oral gavage of 2 g/kg of glucose after 12 hours of fasting. The administered mice were sampled for the blood glucose levels using the test paper at 0, 15, 30, 60, 90, and 120 minutes. The glucose levels of the PCOS group were the highest, suggesting that the delayed glucose clearance in the PCOS group was removed in the YLTB group (**Figure 3A**). Consistently, YLTB significantly attenuated increases in the levels of fasting glucose, serum insulin and HOMA-IR in PCOS mice (**Figures 3B–D**). Meanwhile, the body size of the PCOS group was larger than the control group, assayed by Lee’s index and BMI, while the values of these two indexes were lowered down in the YLTB group (**Figure 3E, F**).

The lipid profiles of the three groups were characterized by determination of levels of total TC, TG, LDL-C and HDL-C. Importantly, the TC levels in the YLTB group was lower than the one in the PCOS group (**Figure 3G**). In contrast, there was no significant changes of TG levels between the PCOS group and the YLTB group (**Figure 3H**). Notably, although the HDL-C levels in the YLTB group was higher than the one in the PCOS group (**Figure 3I**), the levels in the PCOS group were similar to in the control group (**Figure 3I**). On the other hand, LDL-C levels were significantly increased in the PCOS group compared to the control group (**Figure 3J**). However, YLTB had no significant impact on the elevated LDL-C levels in PCOS mice. This indicates that the mechanism by which YLTB acts on TC to affect PCOS is complicated.

### 3.4 YLTB attenuates gut microbiota dysbiosis in PCOS mice

**Figure 4:**
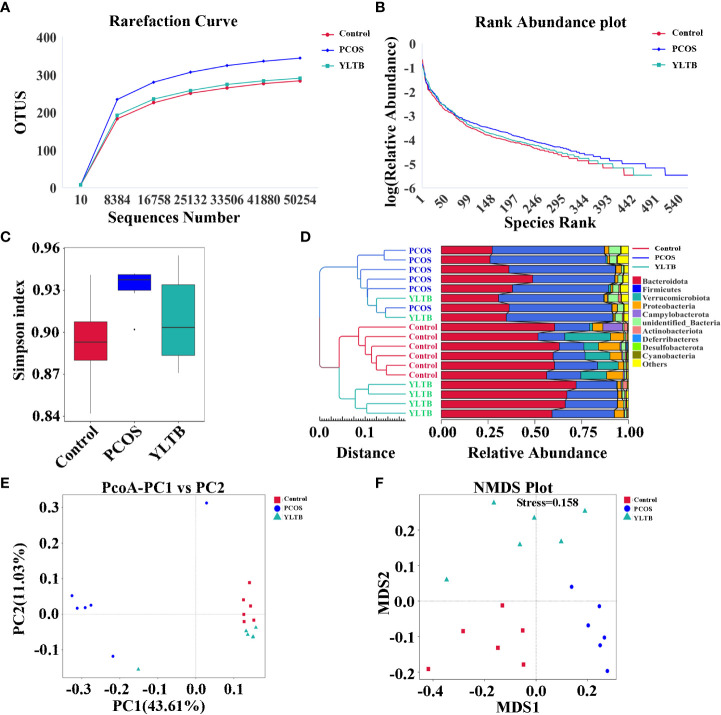
Effect of YLTB on α and β diversity of gut microbiota. **(A, B)** Analysis of gut microbial diversity was performed on the basis of 16S rRNA sequencing and was presented by rarefaction curves and rank abundance curves. **(C)** The α-diversity of gut bacterial assemblages with Simpson index in the mice receiving different treatments. **(D)** Evaluation of β-diversity with bacterial community compositional similarity using UPGMA cluster analysis, and the clustering result and the relative abundance of each sample at the phylum level were displayed. The left side is the UPGMA clustering tree structure, and the right side is the relative abundance distribution map of each sample at the phylum level. **(E, F)** Plots of unweighted UniFrac-based PCoA and nonmetric multidimensional scaling (NMDS) based on Bray-Curtis distance. Each point in the graph represents a sample, the distance between points indicates the degree of variation, and the samples of the same group are represented by the same color. n = 6 mice/group.

Given that the gut microbiota can regulate many metabolites, we next investigated whether the gut microbiota contributes to YLTB manipulation on sex hormone levels and metabolism indicators in mice with PCOS. Before that, the profile of the gut microbiota, simulated by the fecal microbiota, was described for the control, PCOS, and YLTB groups. The 16S rRNA sequencing analysis was performed with fecal samples separately collected from the groups. The rarefaction curves (**Figure 4A**) and rank abundance curves (**Figure 4B**) of the three groups growed in a similar manner, reflecting that the amount of sequencing data and the species richness were qualified for further analysis.

The α diversity indicator Simpson index scored closely for the three groups, showing no difference of species diversity across the three groups (**Figure 4C**). The β-diversity of the gut microbiota of the three groups were also determined using various approaches such as the UPGMA (unweighted pair group method with arithmetic mean) clustering method, principal coordinate analysis (PCoA), and nonmetric multidimensional scaling (NMDS). The weighted unifrac UPGMA clustering method classified the control and PCOS mice samples into two distinct groups (**Figure 4D**), implying that the gut microbial composition of the PCOS mice differed from that of the control mice. Although the YLTB group could not be completely separated from the PCOS group, it was significantly different from the PCOS group, demonstrating that YLTB administration alters the gut microbial composition of PCOS mice (**Figure 4D**). Based on the unweighted unifrac distance, PCoA analysis showed a clear separation among the three groups, with the principal component 1 (PC1) value of 43.61% and PC2 value of 11.03% (**Figure 4E**). In addition, the degree of variation among the three groups was illustrated using NMDS analysis based on Bray−Curtis distance, when stress is less than 0.2 (stress = 0.158, **Figure 4F**). Both PCoA and NMDS analyses revealed that the intergroup distances were greater than the intragroup distances, implying that PCOS and YLTB therapy have an effect on the β-diversity of gut microbiota.

### 3.5 YLTB alters the taxonomic composition of gut microorganism communities at the phylum, genus and species levels

**Figure 5:**
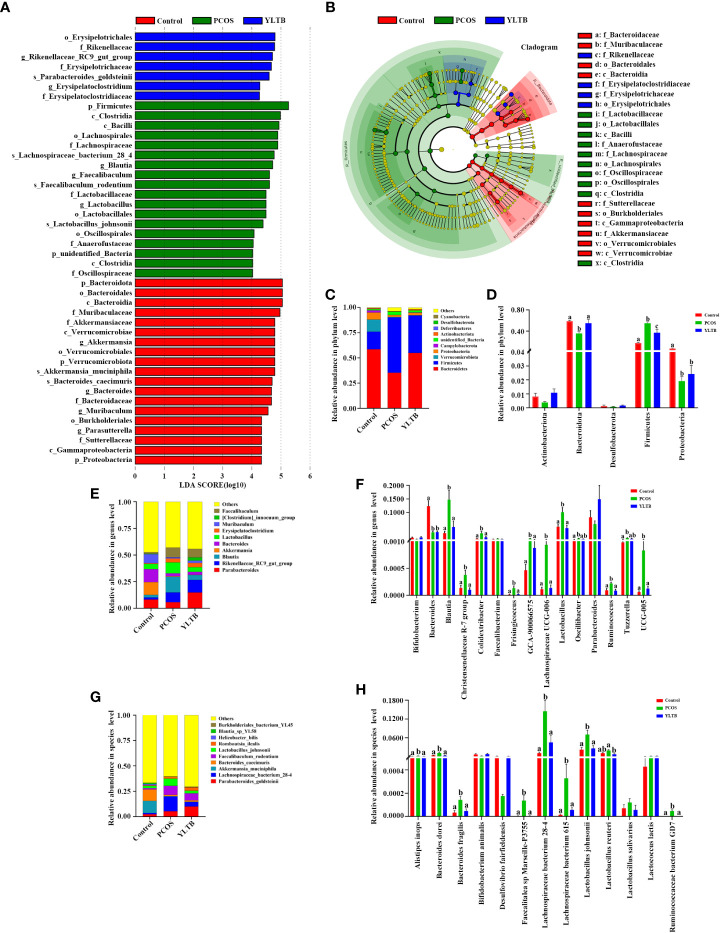
Changes in the taxonomic composition of gut microorganism communities at the phylum, genus and species levels. Statistical differences in the level of biomarkers between the control, PCOS, and YLTB groups were identified using the line discriminant analysis (LDA) effect size (LEfSe) method. **(A)** Taxa enrichment as indicated by discriminant analysis (LDA) scores in the control (red), PCOS (green), and YLTB (blue) treated groups. Only the taxa meeting an LDA significance threshold of four are displayed, and the length of the histogram represents the influence of different species. **(B)** The output of the LEfSe algorithm as visualized by cladograms. Significantly distinct taxonomic nodes are colored and the branch areas are shaded according to the effect size of each taxa. **(C, E, G)** The top ten bacteria with maximum abundance of intestinal bacteria at the phylum, genus and species levels among control, PCOS-treated and YLTB-treated mice. **(D, F, H)** Significant changes in abundance at the phylum, genus and species levels among the control, PCOS, and YLTB groups. N = 6 mice/group. For **D**, **F**, and **H**, statistical significance was determined using one-way ANOVA with Tukey’s multiple comparisons test, and data are presented as the mean ± SEM. a, b and c indicate *P* < 0.05; if 2 groups have the same letter, it indicates no statistical significance.

In addition to diversity details, the abundance of gut microbiota of the control, PCOS and YLTB groups was analyzed, using the linear discriminant analysis (LDA) effect size (LEfSe) method. In our studies, the cut-off threshold of LDA was set at the score 4.0. Our data showed that the *Bacteroidota* phylum was enriched in the control mice, and the *Firmicutes* phylum was enriched in PCOS mice, as well as the *Erysipelotrichales* order in YLTB mice (**Figures 5A, B**). This observation demonstrated a great shift of gut microbiota composition from PCOS mice to YLTB mice.

The detailed differences of the taxonomic composition of gut microbiota among the three groups were shown in the **Figure 5C–H** at the phylum, genus, and species levels. At the phylum levels, *Firmicutes* and *Bacteroidota* were dominant in all three groups (**Figure 5C**). Furthermore, the relative *Firmicutes* abundance levels were highest in PCOS mice, but the levels of *Bacteroidota* and *Proteobacteria* were lower than the ones in other two groups. These changes were attenuated in YLTB mice (**Figure 5D**). For *Bacteroidota*, the *Bacteroides* and *Parabacteroides* genus abundance levels were lower in the PCOS mice than in the control group (**Figure 5E**). For *Firmicutes*, the *Blautia* and *Lactobacillus* abundance levels in PCOS mice were restored to the normal levels by YLTB administration. The similar effects were observed with the abundance levels of *Parabacteroides* (**Figure 5E**). Besides *Blautia* and *Lactobacillus*, *Lachnospiraceae UCG-005* and *UCG-006* were greatly enriched in the PCOS group, which were also significantly reduced by YLTB (**Figure 5F**). *Bacteroides*, *Blautia* and *Lactobacillus* genera were further analyzed for the detailed information at the species levels (**Figures 5G, H**). The data showed that *Bacteroides dorei*, *Bacteroides fragilis*, *Lactobacillus johnsonii* and *Lachnospiraceae bacterium* 28-4 as well as Lachnospiraceae bacterium 615 were enriched in the PCOS group and could be reverted in the YLTB group (**Figure 5H**). These results suggested that YLTB can restore PCOS gut microbiota disturbances.

### 3.6 Association among fecal metabolites, gut microbiota and PCOS mice phenotypes

**Figure 6:**
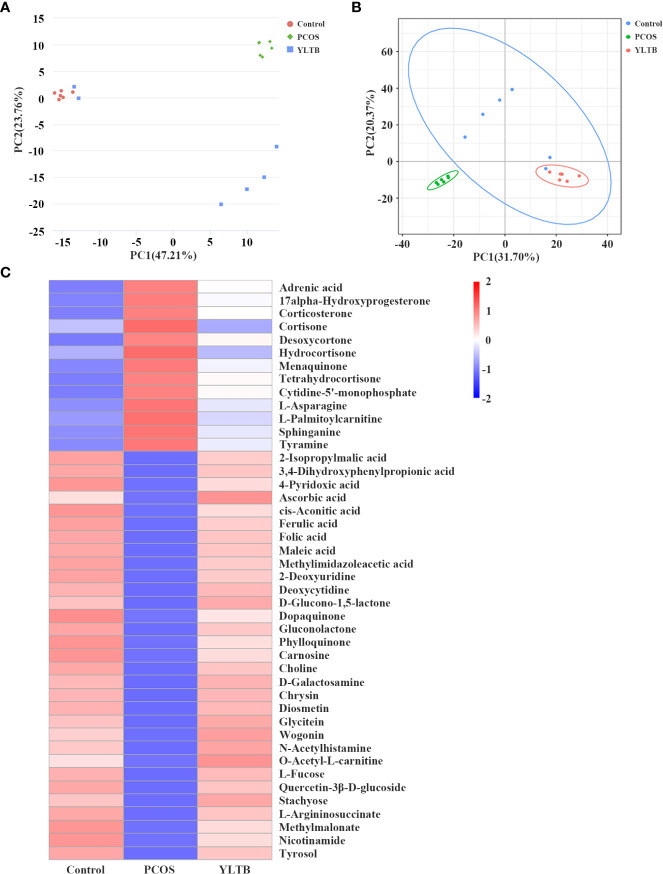
The effect of YLTB on the metabolomic profiles of mouse feces. **(A, B)** Principal component analysis (PCA) and partial least squares discrimination analysis (PLS-DA) were used to observe the overall distribution trends among the three groups of samples. The abscissa PC1 and the ordinate PC2 represent the scores of the first and second principal components, respectively. The scattered points of different colors represent samples of different experimental groups. **(C)** A heatmap of the hierarchical clustering analysis with respect to the relative abundances of 44 fecal metabolites among the three groups. Abscissas represent different experimental groups, ordinates represent different metabolites, and different colors represent the relative expression of metabolites at the corresponding position. N = 6 mice/group.

Since we have shown that YLTB administration greatly changed basal metabolism, ovary dysfunction, and the gut microbiota in PCOS mice, we next seek for more association information among fecal metabolites, gut microbiota and PCOS mice phenotypes using biostatistical approaches. Firstly, the fecal metabolite profile from mice was determined by the untargeted metabolomics. Principal component analysis (PCA) and partial least squares discrimination analysis (PLS-DA) showed a distinct separation of those metabolites among the three groups, with different PC1 and PC2 values (**Figure 6A, B**). The detailed relationship between the metabolites and the three groups were illustrated in **Figure 6C**. 44 fecal metabolites demonstrated that the abundances of fecal metabolites in PCOS mice were different from those in the control mice, which were significantly manipulated by YLTB. These metabolites mainly consist of hormones, vitamins, and organic acids. For example, hormones include cortisone, hydrocortisone, menaquinone, and etc; vitamins such as ascorbic acid, folic acid and phylloquinone, whereas organic acids include cis-aconitic acid, maleic acid, ferulic acid, and etc (**Figure 6C**).

**Figure 7:**
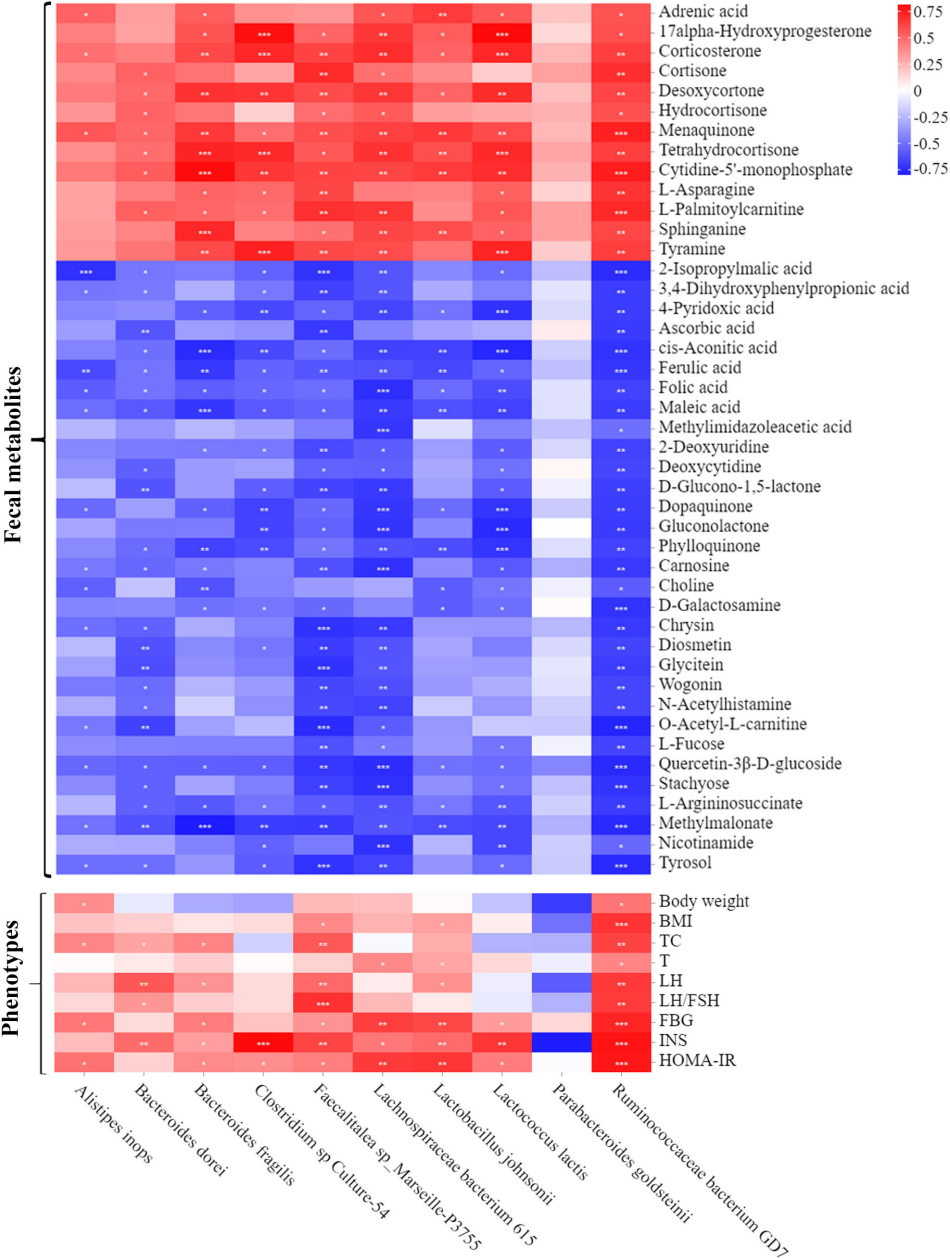
Associations among fecal microbiota, fecal metabolites and PCOS phenotypes. A heatmap of the three-tiered analyses integrating the gut microbiome, fecal metabolites and phenotypes measured by Spearman’s correlation coefficient in the control, PCOS, and YLTB group mice (n = 6/mouse). Associations between the 10 gut microbial species and 44 fecal metabolites are shown in the up panel. The down panel displays associations between 10 gut microbial species and 9 phenotypes. The value range of the correlation coefficient is (-0.75, 0.75). The degree of association is indicated by the color intensity (red represents positive correlations, and blue represents negative correlations). * indicates significant correlation, * *P* < 0.05; ** *P* < 0.01; *** *P <*0.001.

Spearman’s correlation analysis was next used to comprehensively analyze the correlations among fecal metabolites, the gut microbiota and host phenotypes (**Figure 7**). In the relationship between fecal metabolites and the gut microbiota, metabolites (such as ferulic acid, folic acid and methylmalonate) were negatively correlated with 9 bacterial species. Among those metabolites, ferulic acid (FA) was strongly correlated with basal metabolism. The correlations between the gut microbiota and host phenotypes were also shown in the **Figure 7**. Nine bacterial strains (*Alistipes inops*, *Bacteroides dorei*, *Bacteroides fragilis*, *Clostridium sp Culture-54*, *Faecalitalea sp Marseille-P3755*, *Lachnospiraceae bacterium 615*, *Lactobacillus johnsonii*, *Lactococcus lactis*, and *Ruminococcaceae bacterium GD7*) were positively correlated with the host parameters (including body weight, BMI, TC, T, LH, LH/FSH ratio, FBG, INS and HOMA-IR). Taken together, our findings showed a tight association among fecal metabolites, the gut microbiota and host phenotypes in PCOS mice administered with YLTB.

### 3.7 The addition of ferulic acid attenuated ovarian dysfunction in PCOS mice

**Figure 8:**
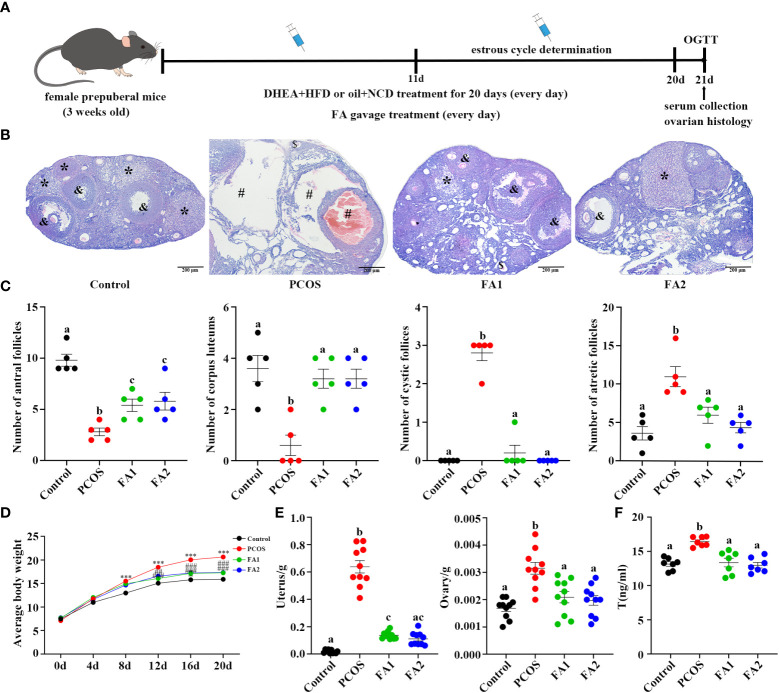
Amelioration of ovarian function in PCOS mice after ferulic acid administration. **(A)** Timeline of the experimental process. **(B)** Changes of ovary stained with Hematoxylin and eosin (H&E) (scale bar=200μm; & indicates antral follicle, * indicates corpora lutea; # indicates cystic follicles, $ indicates atretic follicle). **(C)** Changes in the numbers of antral follicles, corpora lutea, cyst-like follicles, and atretic follicles counted with H&E staining sections (n = 5/group). **(D, E)** Determination of weight of body, uterus and ovaries (n = 10/group, *** P <0.001 compared with the control group, ## P < 0.01; ### P < 0.001 compared with the PCOS group). **(F)** The levels of testosterone in the serum were determined using an enzyme-linked immunosorbent assay kit (n = 7/group). Statistical significance was determined using one-way or two-way ANOVA with Tukey’s multiple comparisons test and data are presented as the mean ± SEM, a, b and c indicate *P* < 0.05, if 2 groups have the same letter, it indicates no statistical significance.

The **Figure 7** showed that FA is a critical metabolite that contribute to the effect of YLTB to the PCOS mice. Therefore, FA was then tested whether it regulates ovarian functions in PCOS mice. The DHEA-treated mice were administered with low-dose FA (FA1 group, 50 mg/kg) or high-dose FA (FA2 group, 100 mg/kg) for 20 days, before they were sampled for further analysis of ovarian function changes (process illustrated as **Figure 8A**). Next, we tested ovarian morphology, body weight, wet weight of the uterus and ovaries and sex hormone alterations among the control, PCOS, FA1 and FA2 groups. H&E staining revealed that the FA administration reduced the number of cystic and atretic follicles, while promoting the development of antral follicles and corpus lutea in the PCOS ovaries (**Figures 8B, C**). The body weight, as well as wet uterine and ovary weight, was significantly reduced in both high and low FA-gavaged mice (**Figures 8D, E**). Consistently, the decreased serum T levels were observed in FA-gavaged mice compared to PCOS mice (**Figure 8F**). However, FA had no impact on the estrus period (**Figure S2C-D**). These findings showed that FA alleviated ovarian dysfunction and reduced pathological damage to ovarian tissues in PCOS mice.

### 3.8 Ferulic acid ameliorates glucose and lipid metabolism disorders in PCOS mice

**Figure 9:**
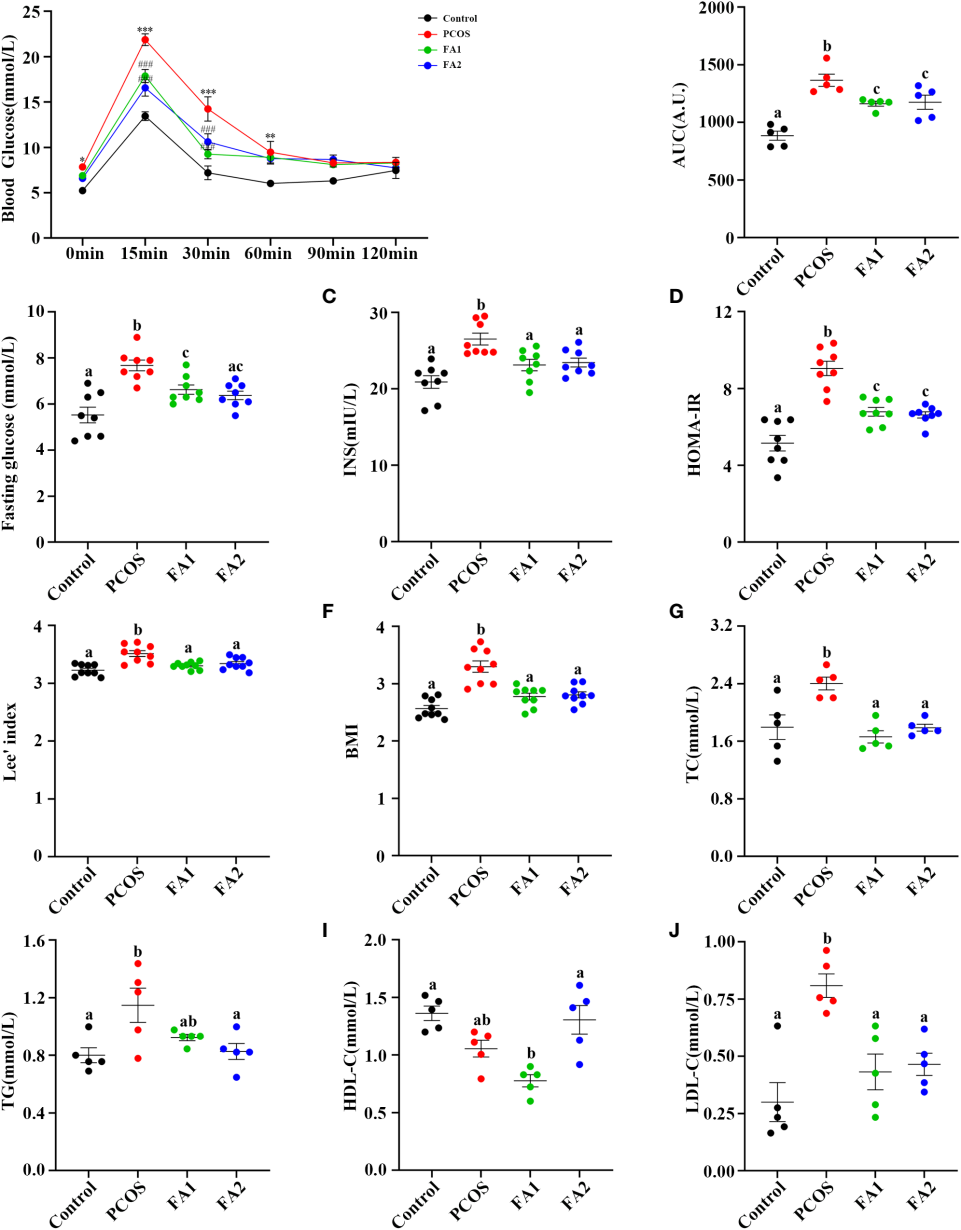
Ferulic acid ameliorated glucose and lipid metabolism disorders of PCOS mice. **(A)** Oral glucose tolerance tests (n = 5/group, ** P < 0.01; *** P < 0.001 compared with the control group, ### P < 0.001 compared with the PCOS group). **(B, C)** Blood glucose and serum insulin (n = 8/group) detection after a 12-hour fast in mice from the control, PCOS, and FA groups. **(D)** Changes of HOMA-IR index. HOMA-IR index = [FBG (mmol/L)] × [FINS (lU/mL)]/22.5 (n = 8/group). **(E)** Lee’s index and BMI calculation (n = 9/group). **(G, H, I and J)** Detection of plasma lipid metabolic indicators, TC, TG, HDL-C, and LDL-C in mice (n = 5/group). Statistical significance was determined using one-way or two-way ANOVA with Tukey’s multiple comparisons test and data are presented as the mean ± SEM, a, b and c indicate *P* < 0.05, if 2 groups have the same letter, it indicates no statistical significance.

FA was next tested for its regulatory effect on disrupted glucose and lipid metabolism in PCOS mice. The **Figure 9A** illustrated that FA administration largely corrected glucose tolerance impairment and dramatically reduced fasting glucose, serum insulin, and HOMA-IR levels in PCOS mice (**Figure 9B–D**). Besides, FA significantly lowered the values of BMI, Lee’s index, TC and LDL-C levels (**Figure 9E, F, G, J**). Consistently, the high FA dose significantly increased the HDL-C levels while decreased the TG levels (**Figures 9H, I**). Differently, the levels of TG and HDL-C in the low dose FA group were lower than the ones in the PCOS group but without statistical differences (**Figures 9H, I**). In general, these studies validated the effect of FA on PCOS similar to YLTB.

## 4 Discussion

PCOS is a common endocrine disorder, which s characterized by ovarian dysfunction, hyperandrogenism and polycystic ovarian morphology. At present, TCM has been used to treat PCOS in Chinese women. The quality control of the YLTB used in this study was investigated by LC-MS (**Figure 1A**). The results showed that the positive and negative ion modes were respectively detected for the top 20 main compounds of YLTB detected, including nobiletin, berberine, quercetin, catechin, etc (**Figure 1B**, **Table 2**, **Table 3**). Numerous studies have found that the main components of YLTB (berberine, catechin, quercetin, and so on) have beneficial therapeutic effects in decreasing the levels of serum T (47, 48), TC (49), LH (48, 49), the ratio of LH/FSH (50), alleviating IR (47, 51, 52), dyslipidemia (51, 53) and other PCOS-related diseases.

With a focus on the therapeutic effects of YLTB on PCOS, our studies demonstrated the complicated association among the ovarian functions, the gut microbiota and metabolites in YLTB-gavaged PCOS mice, using 16S RNA sequencing combined with the non-targeted metabolomics. The ovarian dysfunction in PCOS mice was clearly attenuated by YLTB administration. Consistently, YLTB greatly restored the levels of fasting glucose, serum insulin and total cholesterol from PCOS condition to the normal. Other metabolites such as ferulic acid, folic acid and stachyose were regulated in a similar manner. In the PCOS group, the *Firmicutes* phylum was enriched whereas the *Bacteroidota* phylum was dominant in the YLTB group. Moreover, YLTB suppressed the abundance of the species of *Bacteroides dorei*, *Bacteroides fragilis*, and *Lactobacillus johnsonii* that were all enriched in PCOS mice. The enrichment of these bacteria species was tightly associated with metabolism of many metabolites, as well as host phenotypes in PCOS mice. Among the fecal metabolites, ferulic acid was validated to be effective against PCOS due to its capabilities of reducing ovarian dysfunction, improving disorders of glycolipid metabolism, and associating with the bacterial species.

YLTB ameliorates the host features of PCOS including glucose tolerance, insulin insensitivity, lipid metabolic disorder, and obesity. It is natural to propose that YLTB could substitute current medicines to treat PCOS patients. This idea can be supported by some other TCM studies targeting PCOS (54, 55). For instance, the formula Dang gui shao yao san treatment can significantly reduce plasma LH levels and increase estradiol levels as well as the ovulation rates in PCOS patients (54). Buzhong Yiqi prescription reduces serum androgen levels and regulates lipid metabolism in PCOS patients (55). It is not known yet whether YLTB is better than these two TCM treatments, which might be compared in future. Although ferulic acid has been identified as a critical component, it is not excluded that other possibilities might play important roles.

The therapeutic effect of YLTB on PCOS mice is also related to the amelioration of the gut microbiota dysbiosis. Transplantation with fecal microbiota of healthy control women has been shown to improve PCOS conditions via restoration of gut microbiota dysbiosis. (56). Our data demonstrated that the genus of Blautia, and species of *Bacteroides dorei*, *Bacteroides fragilis* were all enriched in PCOS mice and that YLTB administration attenuated the enrichment of these bacteria species. Consistent with our findings, others reported that *Blautia* was significantly increased in PCOS animals (57) and positively correlated with testosterone concentration, cysts in the ovaries, bodyweight and serum lipids (57, 58). *Bacteroides fragilis* was enriched in PCOS patients, and that clinical indicators such as BMI, T, and LH were positively correlated with it (59–61). In addition, enriched in the intestinal microbiota of PCOS patients, *Bacteroides vulgatus* caused ovarian dysfunction and metabolic disorders in mice (14). Therefore, the present study indicated that YLTB might ameliorate several PCOS disorders in mice by reversing disturbances in the gut microbiota.

Our analysis demonstrated a strong correlation among the fecal metabolites, the host phenotypes and the gut microbiota in PCOS or YLTB mice. FA was prioritized to be investigated for its association with the gut microbiota and PCOS. In our studies, PCOS resulted in a decrease in FA abundance, whereas YLTB reverted it significantly. FA improved disorders of glucose or lipid metabolism in PCOS mice. This is consistent with others’ observations that FA ameliorates lipid profiles and insulin sensitivity (62). Furthermore, our data demonstrated that FA is negatively correlated with 9 bacteria species, all of which were positively correlated with the host phenotypes, such as body weight, BMI, TC,T, LH, FBG, INS and HOMA-IR. Consistently, previous studies shown that FA improves a variety of disorders through modulating gut microbiota (31, 32, 63, 64). Our untargeted metabolomics predicted the roles of some metabolites, and we showed that ferulic acid and the other metabolites (such as folic acid, stachyose, etc.) may have crucial roles, which we will investigate deeply in our in our future studies.

## 5 Conclusion

The current study focuses on the therapeutic effects of YLTB administration on PCOS *via* modulating the gut microbiota and the associated metabolites. YLTB administration clearly attenuated ovarian dysfunction, restored glucose and lipid metabolism, and suppressed the abundance of several bacteria species such as *Bacteroides dorei*, *Bacteroides fragilis*, and *Lactobacillus johnsonii* of PCOS mice. These bacteria were strongly associated with many metabolites. Among the fecal metabolites, the effect of FA against PCOS was similar to that of YLTB.

## Data availability statement

The datasets presented in this study can be found in online repositories. The names of the repository/repositories and accession number(s) can be found below: http://www.ncbi.nlm.nih.gov/bioproject/914098 (accession number PRJNA914098), http://www.ebi.ac.uk/metabolights/MTBLS6736 ( identifier MTBLS6736).

## Ethics statement

The animal study was reviewed and approved by Institutional Animal Care and Use Committee of Chongqing Medical University (Animal Qualification Certificate No.2022109).

## Author contributions

L-JF, QF designed and edited the experiments. Y-NS wrote the manuscript. Y-NS performed the experiments of PCOS model and M-JW conducted data analysis. Y-BD finalized the manuscript. J-PY and X-LW assisted the study. MX and M-HB contributed to experimental design. All authors have read, discussed and approved the final manuscript.

## Glossary

**Table:**

ANOVA	One-way analysis of variance
BMI	Body mass index
CL	Corpora lutea
DHEA	Dehydroepiandrosterone
FA	Ferulic acid
FBG	Fasting blood glucose
FINS	Fasting insulin
FSH	Follicle stimulating hormone
HDL-C	High densitylipoprotein
HFD	High-fat diet
HOMA-IR	Insulin resistance index in the homeostasis model
IR	Insulin resistance
LC-MS	Liquid chromatography–mass spectrometry
LDA	Linear Discriminant Analysis
LDL-C	Low densitylipoprotein
LEfSe	LDA Effect Size
LH	Luteinizing hormone
NCD	Normal chow diet
NMDS	Non-Metric Multi-Dimensional Scaling
OGTTs	Oral glucose tolerance tests
PCA	Principal Component Analysis
PCoA	Unweighted principal coordinate analysis
PCOS	Polycystic ovary syndrome
PLS-DA	Partial least squares discriminant analysis
T	Testosterone
TC	Total cholesterol
TG	Triglycerides
UPGMA	Unweighted Pair-group Method with Arithmetic Mean
YLTB	Yulin Tong Bu Decoction
