# Corrigendum: Effects of Yulin Tong Bu formula on modulating gut microbiota and fecal metabolite interactions in mice with polycystic ovary syndrome

**DOI:** 10.3389/fendo.2023.1184616

**Published:** 2023-04-14

**Authors:** Ya-Nan Su, Mei-Jiao Wang, Jun-Pu Yang, Xiang-Lu Wu, Min Xia, Mei-Hua Bao, Yu-Bin Ding, Qian Feng, Li-Juan Fu

**Affiliations:** ^1^ Department of Herbal Medicine, Chongqing Key Laboratory of Traditional Chinese Medicine for Prevention and Cure of Metabolic Diseases, School of traditional Chinese Medicine, Chongqing Medical University, Chongqing, China; ^2^ Joint International Research Laboratory of Reproduction and Development of the Ministry of Education of China, School of Public Health, Chongqing Medical University, Chongqing, China; ^3^ Department of Physiology, School of Basic Medicine, Chongqing Medical University, Chongqing, China; ^4^ Department of Gynecology, Chongqing Hospital of Traditional Chinese Medicine, Chongqing, China; ^5^ Department of Pharmacology, Academician Workstation, Changsha Medical University, Changsha, China; ^6^ Department of Obstetrics and Gynecology, Chongqing General Hospital, University of Chinese Academy of Sciences, Chongqing, China

**Keywords:** polycystic ovary syndrome, YLTB formula, gut microbiota, metabolites, ferulic acid

**Error in Figure/Table**

In the published article, there was an error in **Figure 3** as published. We recently found by ourselves that the picture 3-J was misplaced. The corrected **Figure 3** and its caption: “The effects of YLTB on glucose tolerance, insulin sensitivity and lipid metabolism in PCOS mice.” appear below.

**Figure 3:**
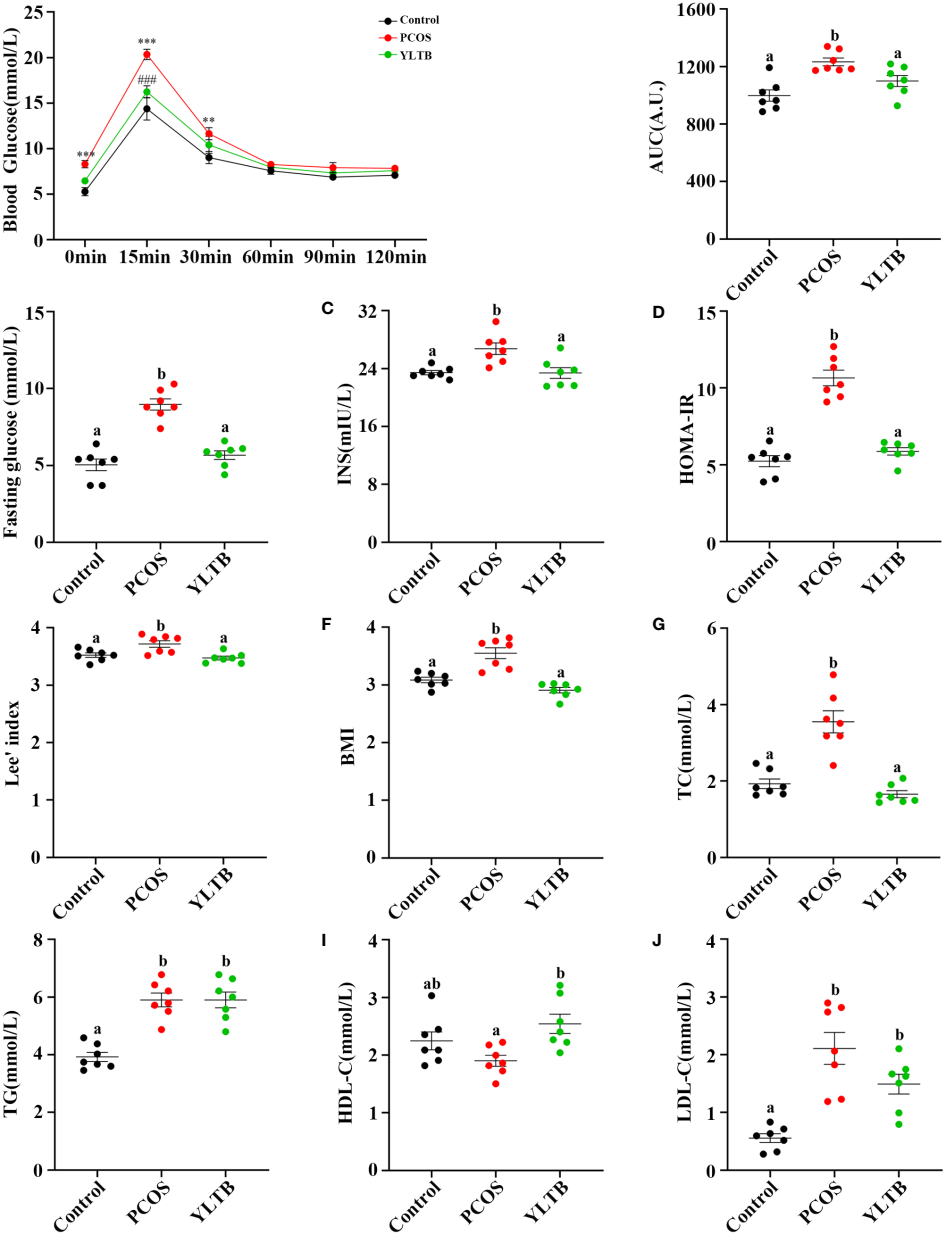
The effects of YLTB on glucose tolerance, insulin sensitivity and lipid metabolism in PCOS mice. **(A)** OGTTs in mice from the control, PCOS, and YLTB groups. The corresponding area under the curve (AUC) values of blood glucose levels in each group were calculated (** P < 0.01; *** P < 0.001 compared with the control group, ### P < 0.001 compared with the PCOS group). **(B, C)** Blood glucose and serum insulin level assessment after 12 h of fasting in mice from the control, PCOS, and YLTB (38.68 g·kg^-1^·day^-1^) groups. **(D)** The homeostasis model assessment of insulin resistance (HOMA-IR) index = [FBG (mmol/L)] × [FINS (lU/mL)]/22.5 in mice from the control, PCOS, and YLTB groups. **(E, F)** Lee’s index = [Body mass (g) × 1,000]^1/3^/body length (cm) and Body mass index (BMI = weight (kg)/height (m^2^) calculation. **(G–J)** Detection of TC, TG, HDL-C and LDL-C to evaluate the level of serum lipid metabolism in mice from the control, PCOS, and YLTB groups. n = 7/group, statistical significance was determined using one-way or two-way ANOVA with Tukey’s multiple comparisons test, and data are presented as the mean ± SEM. a and b indicate P < 0.05; if 2 groups have the same letter, it indicates no statistical significance.

The authors apologize for this error and state that this does not change the scientific conclusions of the article in any way. The original article has been updated.

**Text Correction**

In the published article, there were three errors. There were two unit errors in the **Materials and methods** section and one spelling error in the **Results** section.

A correction has been made to **2 Materials and methods**, 2.2 Liquid chromatography–mass spectrometry, paragraph three. This sentence previously stated:

“injection volume, 2 ml”

The corrected sentence appears below:

“injection volume, 2 μL”

A correction has been made to **2 Materials and methods**, 2.8 16S rRNA sequencing, paragraph four. This sentence previously stated:

“DNA was diluted to 1 ng/L in sterile water according to the concentration.”

The corrected sentence appears below:

“DNA was diluted to 1 ng/μL in sterile water according to the concentration.”

A correction has been made to **3 Results**, 3.8 Ferulic acid ameliorates glucose and lipid metabolism disorders in PCOS mice, paragraph two. This sentence previously stated:

“the high FA dose significantly increased the HDL-C levels while decreased the TC levels.”

The corrected sentence appears below:

“the high FA dose significantly increased the HDL-C levels while decreased the TG levels.”

The authors apologize for this error and state that this does not change the scientific conclusions of the article in any way. The original article has been updated.

**Error in Figure/Table Legend**

In the published article, there was an error in the legend for **Figure 4** as published. 16S rRNA was misspelled as 16S rDNA. The corrected legend appears below.

**Figure 4:**
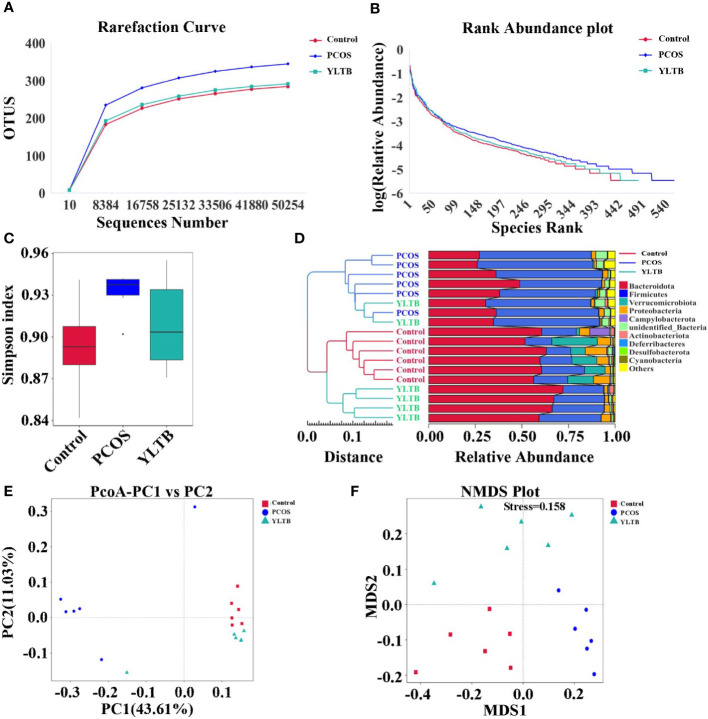
Effect of YLTB on α and β diversity of gut microbiota. **(A, B)** Analysis of gut microbial diversity was performed on the basis of 16S rRNA sequencing and was presented by rarefaction curves and rank abundance curves. **(C)** The α-diversity of gut bacterial assemblages with Simpson index in the mice receiving different treatments. **(D)** Evaluation of β-diversity with bacterial community compositional similarity using UPGMA cluster analysis, and the clustering result and the relative abundance of each sample at the phylum level were displayed. The left side is the UPGMA clustering tree structure, and the right side is the relative abundance distribution map of each sample at the phylum level. **(E, F)** Plots of unweighted UniFrac-based PCoA and nonmetric multidimensional scaling (NMDS) based on Bray-Curtis distance. Each point in the graph represents a sample, the distance between points indicates the degree of variation, and the samples of the same group are represented by the same color. n = 6 mice/group.

“(A, B) Analysis of gut microbial diversity was performed on the basis of 16S rRNA sequencing and was presented by rarefaction curves and rank abundance curves.”

The authors apologize for this error and state that this does not change the scientific conclusions of the article in any way. The original article has been updated.

**Incorrect Supplementary Material**

In the published article, there were two errors in **Supplementary Figure** legend. Figure 1 legend, “20 days instead of 21 days.” The correct material statement appears below.

“(B, C) DHEA or sesame oil were given to female mice for 20 days.”

Figure 2 legend, “FA instead of FC days.” The correct material statement appears below.

“(C, D) Estrus cycles of the control, PCOS and FA groups (n = 5/group).”

The authors apologize for this error and state that this does not change the scientific conclusions of the article in any way. The original article has been updated.

