# Supplementary material for: Effects of Yulin Tong Bu formula on modulating gut microbiota and fecal metabolite interactions in mice with polycystic ovary syndrome
Source: Front Endocrinol (Lausanne). 2023 Feb 6;14:1122709. doi: 10.3389/fendo.2023.1122709 (PMC9939769; doi:10.3389/fendo.2023.1122709)
Supplement: Supplementary file 1 [file DataSheet_1.pdf]

number of figures:2

Supplementary Material

1 Supplementary Data

S1. YLTB(H) has the best effect in improving ovarian dysfunction.

We assessed multiple parameters, such as fasting glucose, testosterone changes, and ovarian morphology, to determine the most effective dose of YLTB on mice ovary functions. The experimental processes were illustrated in Fig. S1A. The estrus cycles of mice in the control group lasts 4 to 5 days, whereas the majority of mice in the PCOS group were in disrupted phase. Treatment with YLTB or metformin did not restore the estrus cycles (Fig. S1B-C). The body weight was significantly reduced in YLTB (H) group than in the PCOS group (Fig. S1D). In comparison to the PCOS group, the YLTB and metformin groups had lower uterus weight trends and significantly lower ovary weight (Fig. S1E). The YLTB and metformin groups dramatically reduced fasting glucose levels compared to the PCOS group, and the YLTB (H) group had the best effect (Fig. S1F). Testosterone levels were lower in the YLTB and metformin groups in comparison to PCOS group (Fig. S1G).

H&E staining and follicle counts were performed to ensure a successful establishment of PCOS model and to determine the effects of YLTB and metformin on ovarian morphology (Fig. S1H). YLTB (H) and YLTB (M) significantly increased antral follicles when compared to the PCOS group. The number of corpus luteum was higher in the YLTB and metformin groups than in the PCOS group. The PCOS group showed more cystic and atretic follicles, while YLTB administration decreased them (Fig. S1I). As a consequence, compared to the YLTB (M), YLTB (L) and metformin groups, YLTB (H) was the most effective in ameliorating ovarian dysfunction.

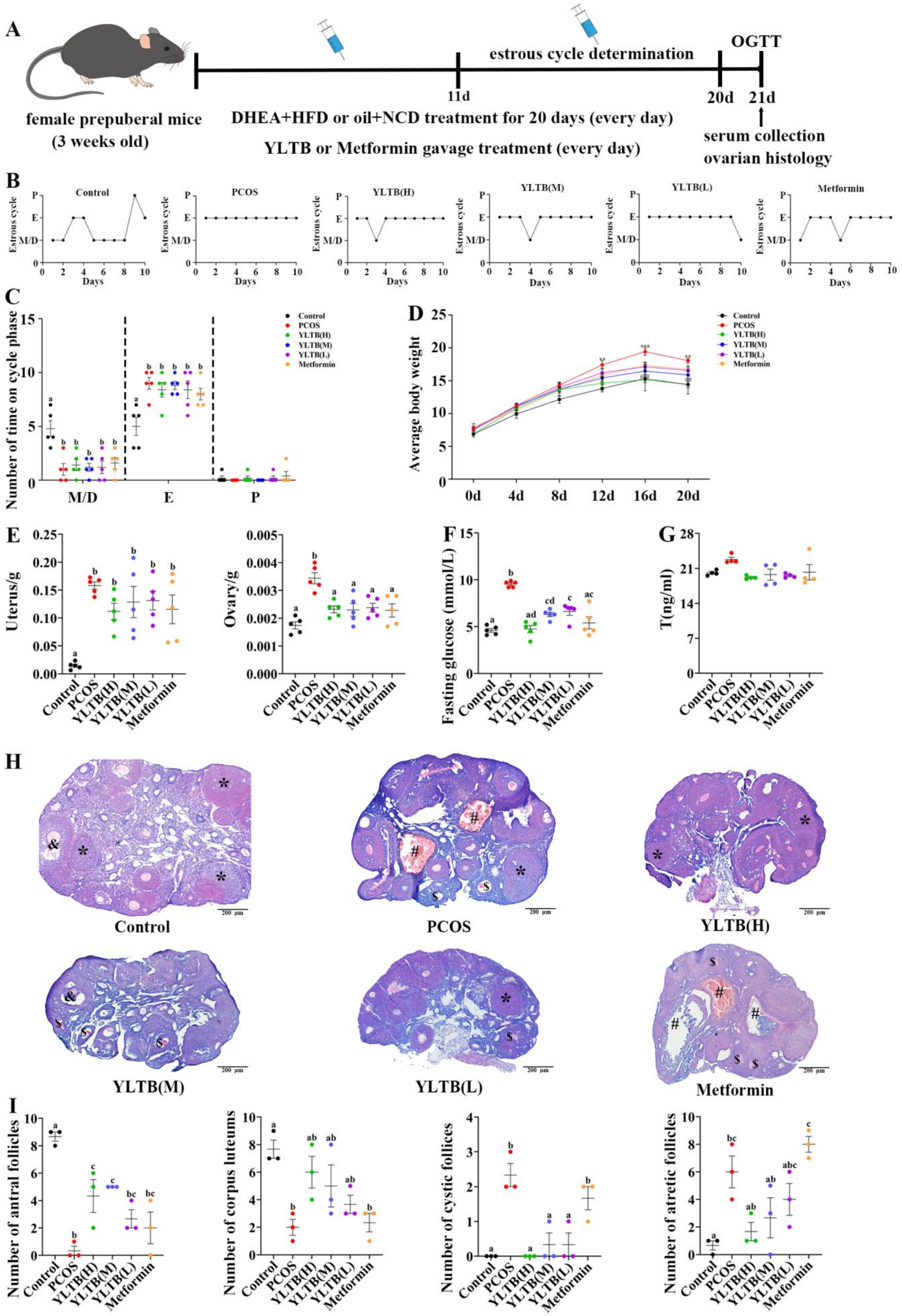
A
estrous cycle determination
OGTT
11d
20d
21d
female prepuberal mice
(3 weeks old)
DHEA+HFD or oil+NCD treatment for 20 days (every day)
YLTB or Metformin gavage treatment (every day)
serum collection
ovarian histology
B
Control
PCOS
YLTB(H)
YLTB(M)
YLTB(L)
Metformin
Estrous cycle
Days
C
Number of time on cycle phase
M/D
E
P
D
Average body weight
0d
4d
8d
12d
16d
20d
E
Uterus/g
Ovary/g
F
Fasting glucose (mmol/L)
G
T(ng/ml)
H
Control
PCOS
YLTB(H)
YLTB(M)
YLTB(L)
Metformin
200 μm
I
Number of antral follicles
Number of corpus luteums
Number of cystic follices
Number of atretic follicles

Figure S1. YLTB(H) has the best effect in improving ovarian dysfunction. **(A)** The timeline of the experimental process. **(B, C)** DHEA or sesame oil were given to female mice for 21 days. At the same time, gavage mice with YLTB and metformin. On the 11th day, record the estrus cycle (n = 5/group). **(D)** Every four days, the body weight of a mouse is measured (n = 5/group, * $P < 0.05$; ** $P < 0.01$; *** $P < 0.001$ compared with the control group, # $P < 0.05$; ## $P < 0.01$; ### $P < 0.001$ compared with the PCOS group). **(E)** The wet weight of a mouse's uterus and ovary was measured (n = 5/group). **(F)** After fasting for 12 h, the blood glucose levels were assessed in mice (n = 5/group). **(G)** Plasma testosterone (N = 4) levels were measured using enzyme-linked immunosorbent assay kit, respectively. **(H, I)** Representative ovarian slices were stained with hematoxylin and eosin (scale bar = 200 µm; & indicates antral follicle, * indicates corpora lutea; # indicates cystic follicles, $ indicates atretic follicle), and the number of antral follicles, cyst-like follicles, corpora lutea, and atretic follicles were counted (n = 3/group). Statistical significance was determined using one-way or two-way ANOVA with Tukey's multiple comparisons test and data are presented as the mean ± SEM, a, b, c and d indicate $P < 0.05$, if 2 groups have the same letter, it indicates no statistical significance.

S2. The effect of YLTB and FA on PCOS mice estrous cycles.

The estrous cycles were measured to investigate the effect of PCOS, YLTB and FA on ovarian functions. Mice in the control group exhibited regular estrous cycles during the 10-day vaginal evaluation, whereas most mice in the PCOS group were in the diestrus phase; YLTB treatment could not restore the estrus cycle (Fig. S2A, B). The estrus period statistics showed that FA had no significant impacts (Fig.S2C, D).

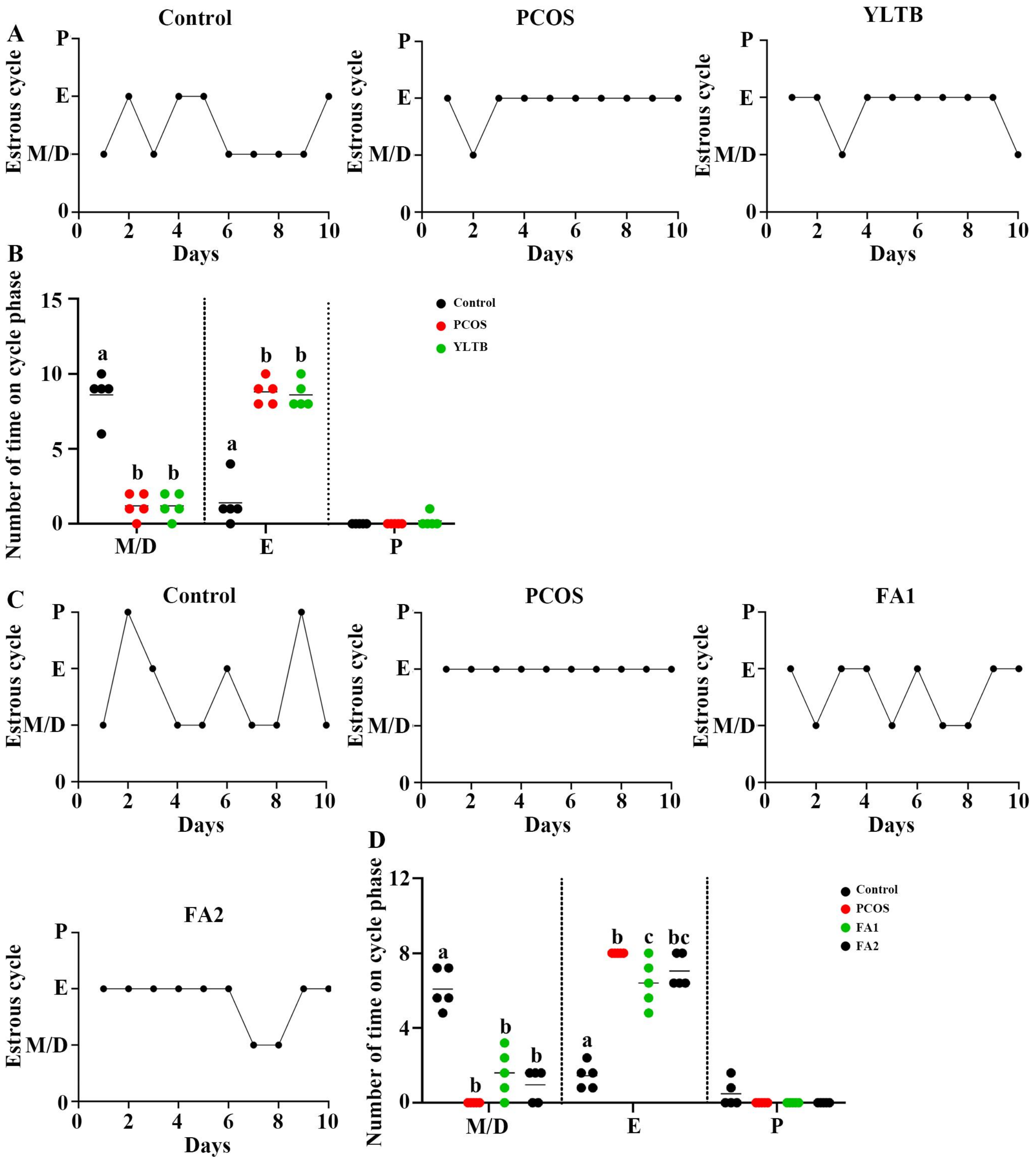

Fig. S2. The effect of YLTB and FA on PCOS mice estrous cycles. **(A, B)** Total of 10 days of assessment of mice estrus cycles among control, PCOS and YLTB (38.68 g·kg^{-1}·day^{-1}) groups (n = 5/group). **(C, D)** Oestrous cycles of the control, PCOS and FC groups (n = 5/group). Statistical significance was determined using one-way ANOVA with Tukey's multiple comparisons test, and data are presented as the mean ± standard error of the mean (SEM). a, b and c indicate $P < 0.05$; if 2 groups have the same letter, it indicates no statistical significance.
